# Compression-coated pulsatile chronomodulated therapeutic system: QbD assisted optimization

**DOI:** 10.1080/10717544.2022.2094500

**Published:** 2022-07-15

**Authors:** Hibah M. Aldawsari, N. Raghavendra Naveen, Nabil A. Alhakamy, Prakash S. Goudanavar, GSN Koteswara Rao, Roja Rani Budha, Anroop B. Nair, Shaimaa M. Badr-Eldin

**Affiliations:** a Department of Pharmaceutics, Faculty of Pharmacy, King Abdulaziz University, Jeddah, Saudi Arabia; b Center of Excellence for Drug Research and Pharmaceutical Industries, King Abdulaziz University, Jeddah, Saudi Arabia; c Department of Pharmaceutics, Sri Adichunchanagiri College of Pharmacy, Adichunchanagiri University, Karnataka, India; d Mohamed Saeed Tamer Chair for Pharmaceutical Industries, Faculty of Pharmacy, King Abdulaziz University, Jeddah, Saudi Arabia; e Department of Pharmacy, School of Medical and Allied Sciences, Galgotias University, Greater Noida, India; f Institute of Pharmaceutical Technology, Sri Padmavati Mahila Visvavidyalayam, Tirupati, India; g Department of Pharmaceutical Sciences, College of Clinical Pharmacy, King Faisal University, Al-Ahsa, Saudi Arabia

**Keywords:** Compression coating, chronobiology, ketorolac tromethamine, PEO WSR coagulant, Eudragit RLPO, quality by design

## Abstract

Pulsatile drug delivery systems have drawn attention in contemporary research for designing chronotherapeutic systems. The current work aims to design pulsatile ketorolac tromethamine tablets using compression coating for delayed delivery with a lag time suitable for the treatment of morning stiffness in arthritis. Rapidly disintegrating core tablets of ketorolac tromethamine were formulated using super-disintegrants, and the optimized formulation was compression using PEO WSR coagulant and Eudragit RLPO for delaying the release. The central composite design and response surface methodology were employed to optimize the formulation and process parameters namely PEO WSR Coagulant (X_1_), Eudragit RLPO (X_2_), and Hardness (X_3_). The dependent variables optimized were lag time and time required for 95% drug release. Analysis using response surface graphs and mathematical modeling of the results allowed identifying and quantifying the formulation variables active on the selected responses. A polynomial equation fitted to the data was used to predict the composition with optimum responses. Compression-coated pulsatile tablets’ optimized composition exhibited a lag time of 9 h and released 95% of the ketorolac tromethamine in 17.42 h. Validation of the mathematical model assured the reliability of QBD in formulation design. *In vivo* X-ray imaging and pharmacokinetic studies established a strong relationship between the coated polymers maintaining the desired lag time for delayed delivery of the active to coincide with the chronobiology for enhanced bioavailability at the right time when needed.

## Introduction

The foremost technical inconvenience faced in the line of the pharma industry is altering the drug delivery patterns as per the therapy requisite (Li et al., 2008). This approach can improve treatment effectiveness, minimize adverse effects, and enhance patient adherence (Songa et al., 2013; Sunil et al., 2013). This technique of modifying drug kinetics and release patterns have beneficial value, particularly in managing several chronic diseases like rheumatoid arthritis that exhibit early morning pathologies. The manifestations of such conditions exacerbate in the prime morning hours as per the circadian rhythm (Buttgereit et al., 2015; Sunil et al., 2011) and require the drug to be available at that time within the therapeutic window to provide relief from the symptoms. This necessitates the drug to be released and delivered quickly within a short duration instantly after the prerequisite lag time (BaHammam et al., 2010; Tinny et al., 2013; Youan, 2010).

Chrono pharmacotherapy effectively manages circadian rhythm disorders (Kalantzi et al., 2009; Politis & Rekkas, 2013). Chronotherapeutic drug delivery systems refer to a treatment method in which in vivo drug availability is timed to match circadian rhythms of disease in order to optimize therapeutic outcomes and minimize side effects. Several techniques have been developed but not many dosage forms for all the diseases are available in the market. Chronotherapeutic drug delivery systems are gaining importance in the field of pharmaceutical technology as these systems reduce dosing frequency, toxicity and deliver the drug that matches the CR of that particular disease when the symptoms are maximum to worse. Finally, the ultimate benefit goes to the patient due the compliance and convenience of the dosage form. Various strategies are available to instigate chronotherapeutic drug delivery that releases the drug in a pulse after a lag time. The compression coating is one of the easy ways to manufacture pulsatile systems owing to less laborious, avoids granulation procedures, enhances drug stability by safeguarding from humidity, and further, it can be simply designed by adjusting the compression instrumentation which is presently used (Gowthami et al., 2021; Hadi et al., 2015). Compression-coated tablets (CCT) consist of active drugs in the inner part that is rapidly disintegrating releasing the drug promptly. This central core is enclosed by the outer coat that does not contain any drug, disintegrates or dissolves very slowly, protects the inner core, and renders a time gap for drug delivery. This technique is beneficial in formulating CCT of distinct features that are appropriate for numerous use (Tang et al., 2018).

Optimizing formulation controls with envied caliber is essential to obtain the required product profile (Jhaveri et al., 2020). The strategy of ‘Quality by Design (QbD)’ is greatly preferred for standardizing the formulation variables (Dalal et al., 2021; Deokar et al., 2021; Naveen et al., 2017). This approach made the optimization process more competent and inexpensive, in contrast to the conventional ‘One factor at a time’ method. QbD method necessitates a few trials to establish the association between distinct factors (Sreeharsha et al., 2020). It renders sound knowledge of the process and aids in achieving the desired product with nominal expenses, time, and manpower (Kurakula & Naveen, 2020). The current study describes the association of the formulation and process variable by the QbD method, following ICH recommendations and employing a statistical software tool.

Ketorolac tromethamine (KT) is an NSAID (non-steroidal anti-inflammatory drug), and a drug of choice in treating the management of rheumatoid arthritis (Vemula & Katkum, 2015). KT, a potent NSAIDs, deploys its effect due to inhibition of both COX-I and II. It is a weak acid, well absorbed from the proximal part of small intestine. KT presents two major problems when administered orally; it causes drastic gastrointestinal side effects as bleeding, peptic ulcer, perforation. Second, it has a short half-life (4–6 h), so it requires persistent administration. Based on these pitfalls various dosage forms of KT have been cited in the literature. KT has been formulated as mucoadhesive hydrogels for pain management after pharmacotherapy. Ahmed A H Abdellatif et al., formulated cyclodextrin/adamantane-grafted polyethylene glycol-based self-assembling constructs for topical delivery of ketorolac tromethamine (Abdellatif et al., 2022). Yet another sustained and controlled systems of KT have been formulated into nanofibrils graft and polymer network hydrogels (Orasugh et al., 2019; Suhail et al., 2021). Priya Shahi et al., attempted to release KT chrono therapeutically by formulating microspheres and tablet in capsule system (Shahi et al., 2015). Therefore, the current work is focused on preparing a novel chronopharmaceutical KT formulation by compression coating using a central composite design.

## Materials and methods

### Materials

KT was a gift sample by Bright Labs, Hyderabad, India. PEO WSR poly(ethylene oxide) polymer coagulant and Eudragit RLPO were procured from Colorcon Asia Pvt. Ltd., Goa, India. Sodium starch glycolate, Microcrystalline cellulose, and lactose were procured from Yarrow Chemicals, Mumbai, India. All Other reagents and chemicals employed were of analytical purity grade and procured from standard manufacturers.

### Formulation development

To formulate CCT of KT, firstly, the immediate release drug core tablets were prepared, and the selected formulation was compression-coated using PEO WSR Coagulant and Eudragit RLPO. Accordingly, concentrations of PEO WSR Coagulant and Eudragit RLPO were optimized using a central composite design.

### Preparation of KT rapid release core tablets

The rapid-release core tablets of KT were formulated by quantifying the drug, diluents together with super disintegrants, and dry binder PVP K30 (Nair et al., 2007). The contents are allowed to pass over #44 mesh to break the lumps and to make the blend uniform. Lubricants and glidants, namely magnesium stearate and talc, were added to the powder mixture and blended in a polybag for 10 seconds (Jacob et al., 2009). The resultant powder mix was compressed with flat punches using an automated nine-station rotary compressor (Rimek Mini Tablet Press-1, Mumbai). The formulation of the core tablets is stated in Table 1.

**Table 1.** Formulation of KT fast-dissolving tablets.

Formulation*	KT	SSG	PVP K 30	MCC
IR-1	20	4	--	Q.s to produce 100 mg tablet
IR-2	20	6	--	Q.s to produce 100 mg tablet
IR-3	20	--	3	Q.s to produce 100 mg tablet
IR-4	20	--	6	Q.s to produce 100 mg tablet

*Each formulation contains 3 mg PVP K 30, 1 mg magnesium stearate, and 1 mg talc.

### Formulation of CCT-KT

CCT-KT was formulated by a press-coating technique based on the design in Table 2. The contents were run over the #44 mesh to break the lumps and make the powder free-flowing. Lactose, PEO WSR Coagulant, and Eudragit RLPO were blended, and further magnesium stearate and talc were added. The blend was then punched directly, where half of the coating mixture was filled into the die, the core tablet was deposited at the midpoint of the die, and then the remaining coating mix was loaded. Compression was done using plain, flat- punches with a rotary compression machine.

**Table 2.** The complete work plan for optimization along with coded and actual values of parameters chosen and constraintsof dependent factors for central composite design.

Selected formulation factors/ Independent variables	Levels			
	−1	+1	−1.681	+1.681
PEO WSR Coagulant (g)-X_1_	100	200	65.9104	234.09
Eudragit RLPO (g)- X_2_	75	150	49.4328	175.567
Hardness (kg/cm^2^)-X_3_	5	8	3.97731	9.02269
Responses / Dependent Variables				Constraint
Lag Time (h)				Target to 9 h
T-95% CDR (h)				Maximum

### Optimization of CCT-KT preparation

The composition of CCT-KT was optimized using response surface methodology and various statistical applications. The formulation parameters chosen were amounts of PEO WSR Coagulant (X_1_), Eudragit RLPO (X_2_), and Hardness (X_3_) as the process parameter at three different levels encoded as −1, 0, and +1. These parameters were optimized for lag time (Y_1_) and time taken to release 95% of KT (T-95%-Y_2_). The constraints for these variables are selected as Target to 9 h [for Y_1_] to achieve required lag time with the compression coating to release the medicament at desired site and Maximum for [Y_2_] to have a prolonged release of KT. Design Expert (V.12, StatEase, Minneapolis, MN, USA) was employed to apply the central composite design that involved 20 investigation trials. Table 2, shows the work plan in terms of coded and actual values of parameters chosen and limits of dependent factors. Entire trial runs were subjected to distinct statistical models like 2FI, and quadratic and compared based on parameters such as relative standard deviation, multiple correlation coefficient (R^2^), predicted, adjusted R^2^values, and the ideal model was opted (Maltesen et al., 2008). The polynomial equations were developed to express the relationship between the selected independent and dependent variables and were validated statistically using ANOVA. Quadratic regression was applied to quantify the response in each experiment, and an investigation was conducted.

$${Y_{i(\mathrm{Quadratic})}=b_{0}+b}_{1}X_{1}+b_{2}X_{2}+b_{3}X_{3}+b_{4}X_{1}X_{2}+b_{5}X_{1}X_{3}+b_{6}X_{2}X_{3}+b_{7}X_{1}^{2}+b_{8}X_{2}^{2}+b_{9}X_{3}^{2}$$

Where,Y_i_ – Selected response or dependent variable,b_o_ – Arithmetic responseb_i_ – Estimated coefficient for main effects (X_1_, X_2_, X_3_); interaction terms of main effects (X_1_X_2,_ X_2_X_3_, X_1_X_3_) and polynomial terms of independent variables (X_1_^2^, X_2_^2^, X_3_^2^)

### Evaluation of prepared CCT-KT tablets

The physicochemical characteristics of the core tablet and CCT-KT were studied. The dimensions and tablet hardness (*n* = 3) were estimated by an automated hardness tester (Mitutoyo Hardness Tester, Mitutoyo South Asia Pvt. Ltd., Delhi, India) (Hassan, 2007; Naveen et al., 2020a). The friability (%, Roche Friabilator, Electrolab, Mumbai, India) and time required for the core tablet to disintegrate (Disintegration tester USP, ED-2AL, Electrolab, Mumbai, India) were estimated (*n* = 6) (Nair et al., 2022). Uniformity of weight of the tablets (*n* = 20) and drug content was estimated. To check the drug content uniformity of each preparation, 10 tablets were triturated; the powder quantity equivalent to 50 mg of KT was dissolved in phosphate buffer (pH 7.4) solution. The solution was filtered and diluted suitably, and the drug content was estimated using RP- HPLC method reported earlier (Vemula & Katkum, 2015).

### Determination of swelling index

To know the swelling behavior, the weight (*W*_1_) of the dry tablet was noted and then immersed in a glass jar with 200 mL 0.1 N HCl. It was stirred at a rate of 200 rpm on a magnetic stirrer (Remi magnetic stirrer, Remi Instruments, Mumbai, India) (Dorozyński et al., 2004; Tadros, 2010). The tablets were withdrawn at regular intervals and the exterior of the tablet was portion was moped with filter paper to get rid of surplus water. The study was performed for 2 h, and the tablets were weighed (*W*_2_). The percentage swelling index was calculated using the equation; Swelling index (%)= W_2_-W_1_/W_1_ * 100 (Sree Harsha et al., 2019).

### Drug release study

*In vitro* drug release studies (*n* = 3) were conducted for CCT-KT with USP type II apparatus (Electrolab Dissolution Tester, Electrolab, Mumbai, India) containing 20 mg of KT (Chaudhary et al., 2021). The dissolution media was stirred at a rate of 50 rpm in a 900 mL medium (0.1 N HCl for an initial 2 h; 5.5 pH buffer for the next 4 h and then in phosphate buffer pH 7.4) maintained at 37 ± 0.5° C. Dissolution samples 5 mL were withdrawn at regular time intervals and replaced with fresh medium maintained at the same temperature to maintain the sink conditions. The dissolution samples were filtered and analyzed for the content of KT dissolved using the RP-HPLC method mentioned earlier (Talukder & Fassihi, 2008).

### Surface morphology

Scanning electron microscopy (SEM, Zeiss, EVO 18, Carl Zeiss SMT Ltd., UK) micrographs of the tablet with optimized composition were taken to analyze the surface morphology. The tablet was subjected to a disintegration test for 5 h and 10 h, after that, the tablet was removed and dried at 40 °C in a hot air oven for one day before being evaluated for topographical features (Reddy et al., 2014). The samples were positioned on a copper stump with double sticky tape and then studied at an increasing potential of 15 kV.

### Standardization and validation of optimization outcome

Design-Expert software (V.12, StatEase, Minneapolis, MN, USA) was employed to generate the experimental model, mathematical relationships, and response surface graphs. Using a mathematical model, an optimum formulation composition was decided based on the desirability of the response (Naveen et al., 2020b). The response surface graph elucidated the relationship between the independent and dependent parameters. ANOVA studied the influence of various factors on the slope coefficients. As a part of design validation, the relative uncertainty was enumerated by using the dissimilarity of predicted and experimental values.

### Compatibility studies

FTIR spectra of pure drug KT and powdered tablet preparation were recorded using Shimadzu FTIR 8300 Spectrophotometer (Shimadzu, Tokyo, Japan). Dry KBr was added to the samples, the mixture was placed in the sampler, and a diffuse reluctance IR spectrum was obtained (Nair et al., 2020).

### Stability studies

As recommended by ICH, stability testing was carried out for the formulation with optimized composition. The CCT-KT tablets were packed in aluminum strips having an inner coat of polyethylene and stored at 40 ± 2 °C and 75 ± 5% RH in the stability chamber for 6 months (Chaudhary et al., 2011). After storing for 6 months, the samples were tested for the drug content and *in vitro* dissolution profile (Krishnaiah et al., 2003). Similarity factor for dissolution rate was compared.

### In vivo studies

Animal used: New Zealand White rabbits

Sex: Male

Age and Weight: 2.5 kg

*In vivo* studies were conducted after obtaining approval from the animal ethical committee of the institution (SACCP-IAEC/2020-21/18, dated; 13/11/2020).

#### In vivo imaging for analyzing residence time

*In vivo*, X-ray imaging was performed to validate the formulation’s transit through the gastrointestinal tract(GIT). The animals have fasted overnight before the study. Abdominal X-ray imaging was carried out to confirm the nonexistence of any other radio-opaque material.

In the final optimized formulation, KT was replaced with radio-opaque material (Barium sulfate) to visualize the tablet under X-ray. The tablet was administered using an endotracheal tube with sufficient water. Total tablet weight is adjusted concerning the animal body weight. After administration, radiographic images were captured at various time intervals to locate the location of the tablet and its disintegration (2 h and 10 h) (Naveen et al., 2018; Tang et al., 2018).

#### Pharmacokinetic studies

Twelve rabbits were selected and divided into two groups of six animals each (Group I- Drug solution- Control group and Group -II- Test group- Optimized formulation). Before the initiation of the experiment, all the animals were kept on adaptation for seven days. Animals were fasted overnight before administering control and test samples. KT drug solution (10 mg/kg) was administered orally using a feeding tube. Correspondingly, the test formulation’s 6.59 mg/kg dose was given to group-II animals. Blood samples (0.5 mL) were collected at various time intervals (0.5, 1, 2, 3, 4, 6, 9, 12, 15, 18 h) from the ear vein in heparinized tubes. Plasma was separated by centrifugation of blood for 10 min at 5000 rpm and analyzed for drug concertation using the RP-HPLC method adopted from the literature (Sohail et al., 2015).

## Results and discussion

### Physicochemical properties of core tablets

The prepared core tablets have shown uniform thickness and diameter (Table 3). Hardness was maintained in the range of 102.5 ± 2.05 N. The percentage weight variation was found to be within the acceptable ranges. The percentage friability was found to be <1%. All the prepared batches were passed for the weight variation, as portrayed in Pharmacopeia.

**Table 3.** Physicochemical properties of core tablets.

Formulation	Thickness (mm)	Diameter (mm)	Weight variation (%)	Drug content (%)
IR-1	3.36 ± 0.01	5.29 ± 0.01	<10	98.74 ± 0.47
IR-2	3.35 ± 0.02	5.30 ± 0.02	<10	99.21 ± 0.21
IR-3	3.36 ± 0.02	5.29 ± 0.02	<10	99.34 ± 0.19
IR-4	3.37 ± 0.01	5.29 ± 0.01	<10	99.54 ± 0.12

**Figure 1.**
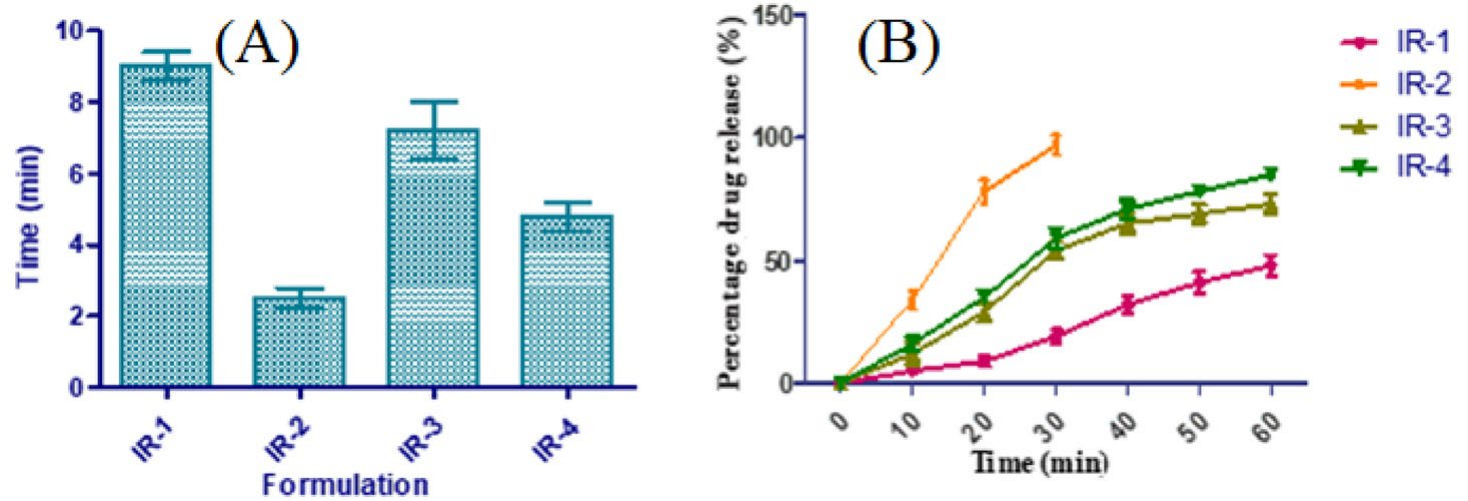
Disintegration (A) and drug release profiles (B) of core tablets of KT.

The impact of selected super disintegrants on disintegration and drug delivery pattern was also observed. As depicted in Figure 1, IR-2 containing 6 mg of SSG demonstrated the least disintegration time owing to its quick water adsorption and swelling that results in rapid disintegration. This was further supported by the drug release pattern. *In vitro* release profile was studied for all the immediate-release tablets. Amidst all, SSG was noted to possess good release in contrast to others, and a rise in the concentration of SSG from 4 to 6 mg has exhibited an improvement in the release with a starting burst release of 34% at 5 min and 86% and 78% at the end of 10 min. Additionally, the incorporation of PVP K30 may boost the solubility and dissolution of poorly soluble drugs and enhance the solubility of KT (Khan et al., 2013; Shah et al., 2009). Though the IR-2 tablet disintegrated within 2 min, it took almost 30 min for the complete release of KT. The variation in the disintegration and dissolution characteristics of SSG were ascribed to alterations in cross-linking range and extent of carboxymethylation (which causes hydrophilicity by distorting the hydrogen bond and permits water entry to the molecules). By considering all the discussed results, IR-2 formulation was selected as a core material to design CCT-KT.

### Optimization of CCT-KT preparation

The central composite design was used to determine the optimum level of the factors considered and their relationship with the time gap before KT release. Table 4 summarizes the results of 20 different experimental runs. The lagging time of all the trail preparations was observed between 2.3 to 10.2 h, while T-95% CDR was found to be in the range of 5.8 and 17.6 h. The results were analyzed for the influence of selected independent variables using statistical model f value and ANOVA.

**Table 4.** Projected trial batches and their evaluated responses as per central composite design.

		Factor 1	Factor 2	Factor 3	Response 1	Response 2
Std	Run	A:PEO WSR Coagulant	B:Eudragit RLPO	C:Hardness	Lag Time	T-95% CDR
		mg	Mg	kg/cm2	H	h
16	1	150	112.5	6.5	6.8	13.2
5	2	100	75	8	4.4	13.2
2	3	200	75	5	6.2	9.8
20	4	150	112.5	6.5	6.5	12.9
7	5	100	150	8	5.9	13.5
18	6	150	112.5	6.5	6.6	13.2
15	7	150	112.5	6.5	6.4	12.8
8	8	200	150	8	10.2	17.6
3	9	100	150	5	4.2	7.4
4	10	200	150	5	7.2	13.4
13	11	150	112.5	3.97731	4.8	6.3
1	12	100	75	5	2.5	5.8
10	13	234.09	112.5	6.5	8.4	15.2
19	14	150	112.5	6.5	6.7	13.4
6	15	200	75	8	9.2	15.9
11	16	150	49.4328	6.5	5.1	11.3
14	17	150	112.5	9.02269	7.9	19.1
17	18	150	112.5	6.5	6.5	13.1
12	19	150	175.567	6.5	6.8	13.9
9	20	65.9104	112.5	6.5	2.3	7.7

For all the responses quadratic model was opted, depending on the sum of squares (Type I) and fit summary (adjusted and predicted R^2^) (Table 5). A quadratic model was chosen (highest order polynomial), where the auxiliary terms are notable and the model is not aliased. The predicted R^2^ for both the responses; 0.9075 and 0.9182 were in close agreement with the adjusted R^2^ of 0.9746 and 0.9780, respectively. Thus, confirming the efficiency of the model to run the design space. The Model F-value of both the responses was found to be 81.87 and 95.04 inferring that the model is appropriate. There is just a 0.01% probability that such a great F-value might occur due to noise.

**Table 5.** Model Summary Statistics of selected responses.

	Source	Sequential *p*-value	Lack of Fit *p*-value	Adjusted R²	Predicted R²	Remarks
Lag Time	Linear	< 0.0001	0.0034	0.9376	0.9119	
	2FI	0.3153	0.0033	0.9409	0.9204	
	Quadratic	0.0092	0.0187	0.9746	0.9075	Suggested
	Cubic	0.4432	0.0055	0.9754	−0.3972	Aliased
T- 95% CDR	Linear	< 0.0001	0.0014	0.9371	0.9110	
	2FI	0.1579	0.0018	0.9474	0.8897	
	Quadratic	0.0079	0.0113	0.9780	0.9182	Suggested
	Cubic	0.0044	0.3602	0.9961	0.9531	

**Figure 2.**
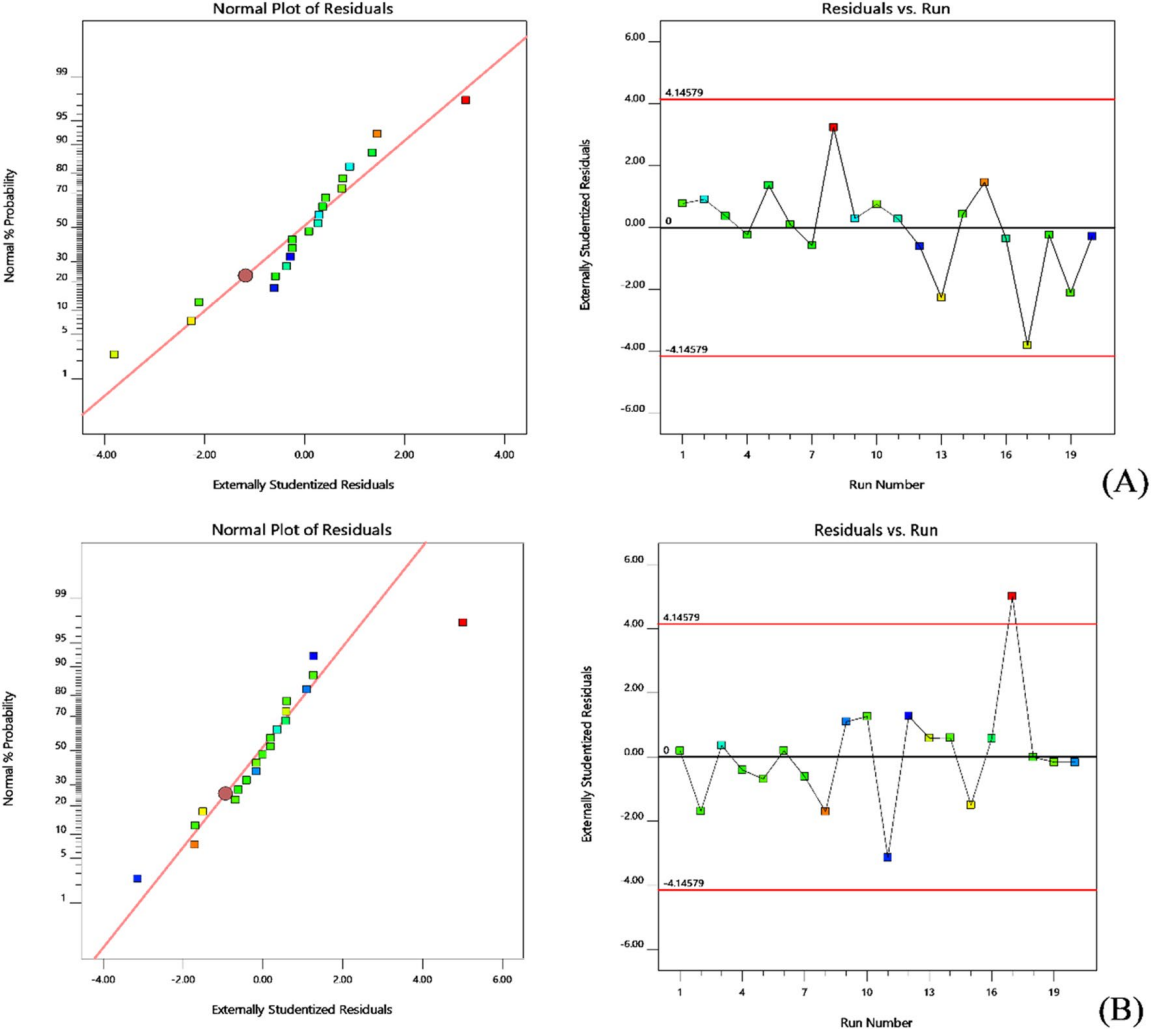
Normal probability plots and model residuals Vs. test orders of the residuals for A) Lag time and B) T-95% CDR.

A good correlation was observed between the experimental and predicted data while representing the two experiment responses. The probability plot ensures that the residuals are inside the normal distribution, i.e. they are all straight lines (Figure 2A and B). The favorable measure of the plot is by exterior studentized residuals while other raw or inner studentized residuals aren’t recommended. In addition, a general residual plot (Studentized residuals Vs normal % probability) was employed to measure the adapted models and to ensure model accuracy (Sarathchandiran et al., 2019). Further, the effect of test orders on the adapted design was illustrated by model residuals versus test order (Ahmed et al., 2015). The external studentized residuals had a straight-line distribution with a small deviation in this study, indicating that the model adopted was statistically admissible (Figure 1A) (Singh et al., 2011). Figure 1B, depicts experimental runs set against the residuals, indeed a working method to recognize the slinking variables which may alter the study results. An arbitrary distribution pattern is noted in the plot that denotes time-coupled variables lurking in the framework.

The model repeatability can be assured with the value of the coefficient of variation (CV). The reproducibility of the present model was found to be CV < 10%. Relatively low CV values were noted in the study which ensures model accuracy and reliability. Insufficient fit can result in an ineffective model to represent the complete data. Therefore, lack of fit is a prerequisite to determining that the equations developed by the model are coherent in forecasting the responses. All the *p* values of PS, EE, and SI were observed as insignificant and so the model chosen was appropriate (Zhang et al., 2020).

The inference of quantitative impacts of the fact components was investigated using ANOVA. Multiple regression analysis was used to derive polynomial equations from the data. The ANOVA findings outperformed the quadratic equation’s statistical significance, and the *p*-value for the significance of model terms was <0.0500. The test design stipulated that lag time was greatly affected by a) antagonist effect of A with a *p*-value of 0.0016 and b) synergistic effect of A, B, C, and AC with a *p*-value of <0.0001, <0.0001, <0.0001and <0.0228 correspondingly, with inflated A effects (Equation 1). Response 2 was profoundly contrived by i) antagonist effect of polynomial term of A with a *p*-value of 0.0013 and ii) synergism effect of A, B, C, and AB with a *p*-value of <0.0001, 0.0001, <0.0001, and 0.0442, respectively, and among all the significant variables, term C effect the EE with high enormity (Equation 2). The equations obtained from the responses for the best possible model were mentioned below:

(1) $$\text{Lag}\;\text{Time}\;=+6.57+1.91\text{ A }+0.5901\text{ B }+1.08\text{ C }-0.1500\;\text{AB}\;+0.3000\;\text{AC}\;-0.0250\;\text{BC}\;-0.3550{\;A}^{2}-0.{1429\;B}^{2}-0.00{14\;C}^{2}$$

(2) $$T-95\%\;\text{CDR}\;=\;+13.10\;+2.15\text{ A }+0.8474\text{ B }+3.32\text{ C }+0.425\;\text{AB}\;-0.4000\;\text{AC}\;-0.4000\;\text{BC}\;-0.60{94\;A}^{2}-0.20{28\;B}^{2}-0.{1675\;C}^{2}$$

**Figure 3.**
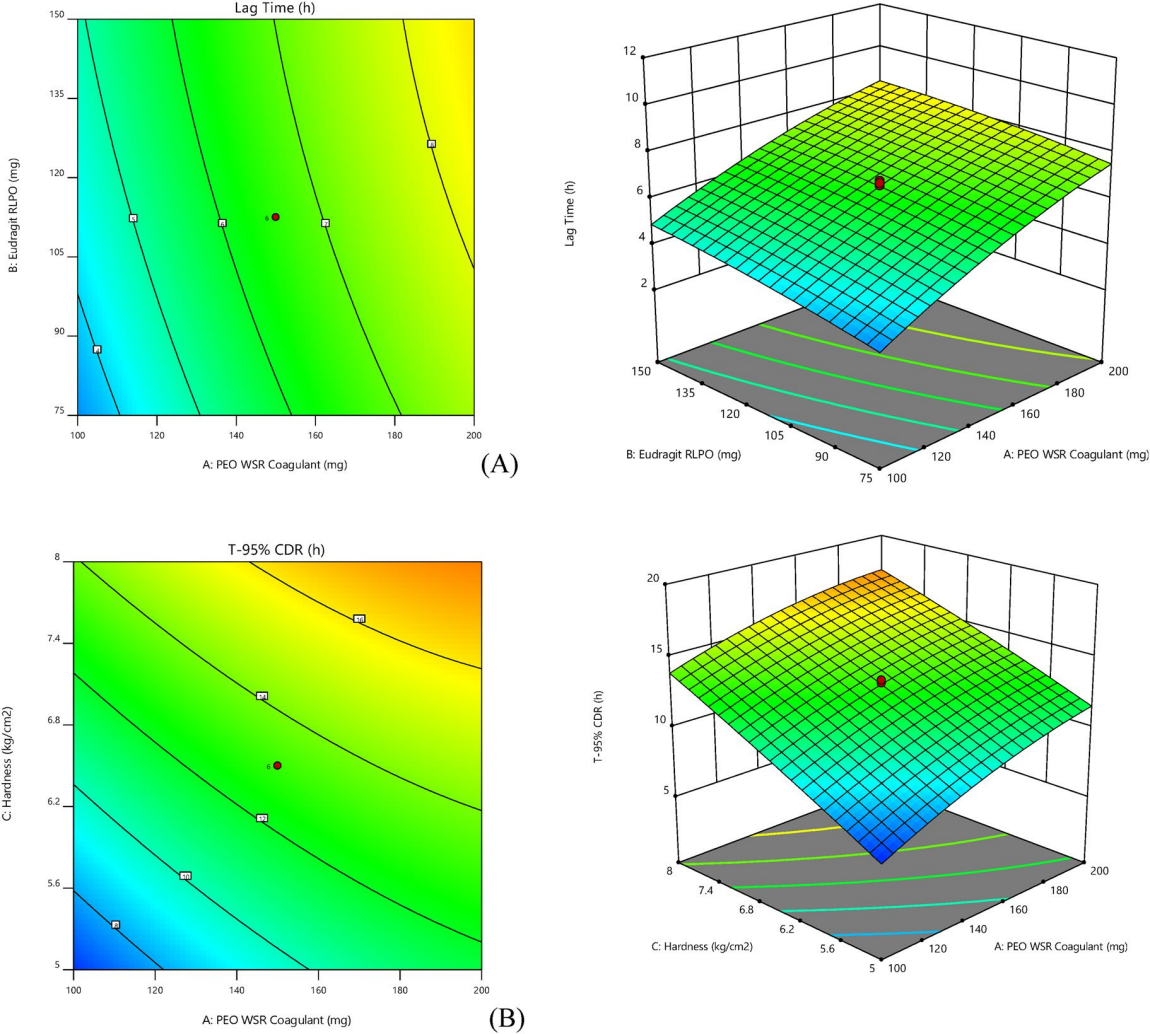
Response surface [contour] graphs and 3-Dimensional plots for lag time (A) and T-95% [B] (contour and three-dimensional).

ANOVA co-efficient with their *p* values for both the responses were shown in Table 6. Obtained results were employed to estimate the significance of the model coefficients. Further, the influence of individual variables on responses was analyzed and interpreted by RSM (Singh et al., 2011). Figure 3 depicts the relationship of selected responses with the variables, in 2 D and 3 D contour plots. RSM was employed to interpret the effect of independent variables against the obtained individual responses. 3 D response surface graphs are crucial to illustrate the interaction and main effect. The obtained responses are visualized employing contour plots (Adetunji & Olaniran, 2020).

**Table 6.** ANOVA coefficients table for both the responses with *p*-value. (Values in bold type indicate significant factors).

	Intercept	A	B	C	AB	AC	BC	A²	B²	C²
Lag Time	6.57	1.91	0.59	1.09	−0.15	0.3	−0.025	−0.36	−0.14	−0.0014
*p*-values		**< 0.0001**	**< 0.0001**	**< 0.0001**	0.21	**0.0228**	0.8273	**0.0016**	0.1166	0.9866
T-95% CDR	13.10	2.16	0.85	3.32	0.43	−0.4	−0.4	−0.61	−0.20	−0.17
*p*-values		**< 0.0001**	**0.0001**	**< 0.0001**	**0.0442**	0.0555	0.0555	**0.0013**	0.1712	0.2515

**Figure 4.**
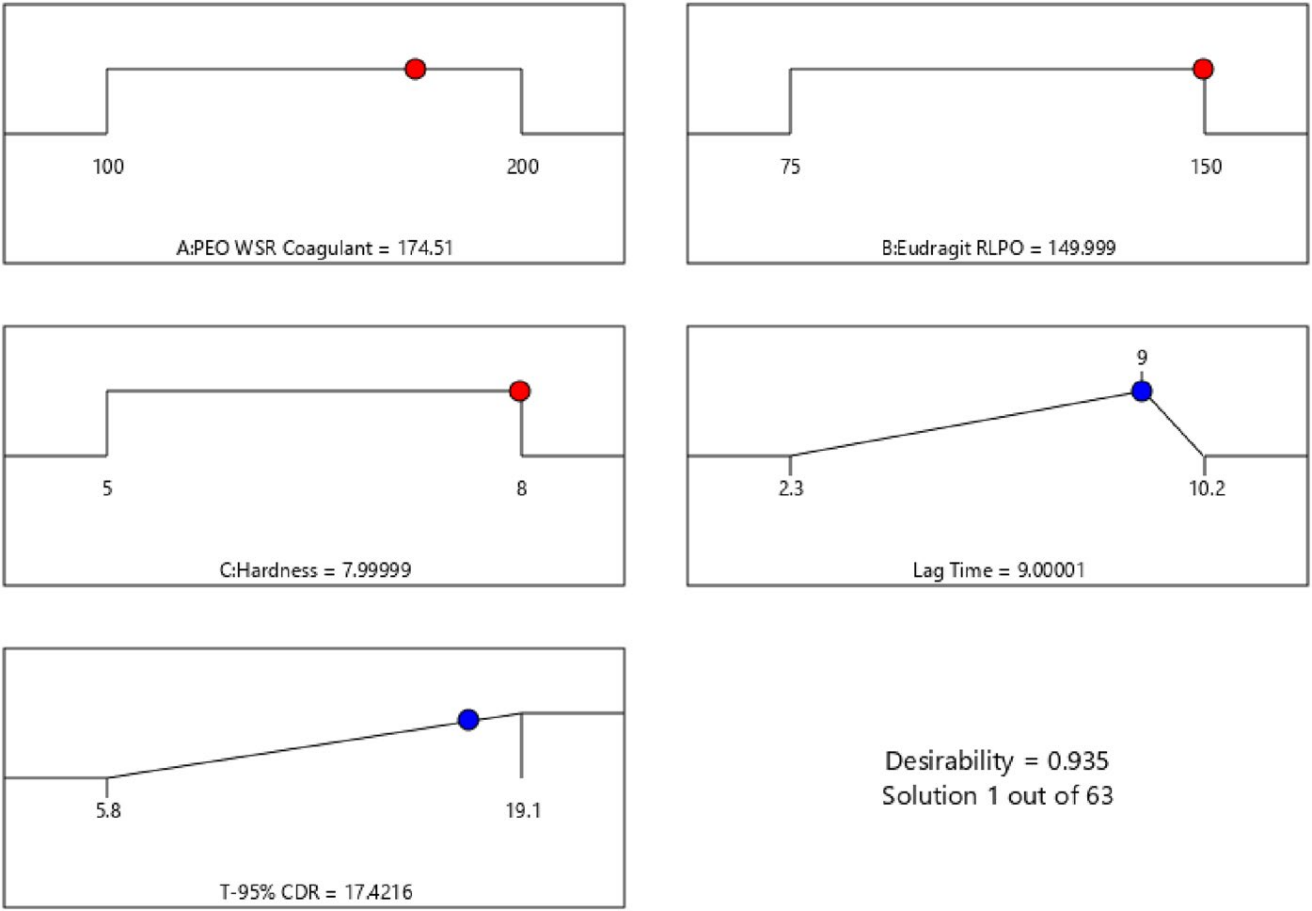
Desirability plot in optimizing the selected factors to achieve the desired responses.

The desirability function (D) was employed to standardize the model’s order obtained by statistical analysis. Every response was laid a limit (lag time to 9 h and T-95% CDR to a minimum) to draft an inlay graph to enhance the independent variables. All the 3 feasible independent variables were included in the design for standardization. The independent variables (optimal level) signified a maximum of 0.935 D (Figure 4) value for both responses in the desirability function plot. Hence, execution of this setting helps in achieving the desired lag time of 9 h and T-95% CDR of 17.42 h. By using these, optimized concentrations, the CCT-KT formulation was formulated and validated the study design. Relative uncertainty was observed below 2%, which ensures design accuracy.

### Swelling study

A swelling study was employed to measure the gelation capacity of the polymer with regard to time and its effect on the drug release from the core tablet. The swelling index value of optimized preparation was determined at various time intervals. The response indicates that the high concentration of the PEO and Eudragit RLPO polymer, enhanced the swelling time and also has a high swelling index. Optimized formulation exhibited swelling within 10 min with a low swelling index, whereas swelling index was increased up to 60 min, showed the highest swelling index of 49.26%, and took more time for absolute swelling. After absorbing an adequate amount of water, the polymer eroded from the tablet surface and correspondingly the swelling indices decreased (after 1 h). This ability of hydration is a requisite for the preparation to regulate the time wrap of drug release.

### In vitro drug release

**Figure 5.**
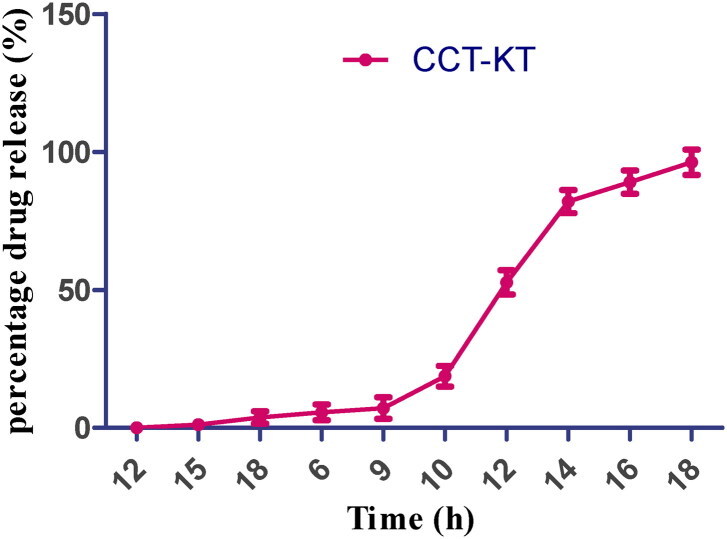
*In vitro* drug release profile of CCT-KT. (Values represent the mean of 6 determinations; *n* = 6, Avg ± SD).

The *in vitro* drug release was performed for all the preparations (*n* = 6). The dissolution profile is depicted in Figure 5. The optimized preparation exhibited an optimum time gap of 9 h following which there was an immediate drug release that reached 52.68%, within 12 h and more than 95% at the end of 17 h. PEO is a non-ionized hydrophilic polymer that absorbs water 7 folds of its original weight. Its swelling is independent of the pH, it can be employed in designing several pulsatile or controlled drug delivery systems. Even though other grades of PEO have a higher gelation index, the gel formed was eroded easily due to its low strength, but the gel formed for CCT prepared with PEO WSR coagulant was sturdy and did not erode easily. The observed gel power was related to the viscidity and weights of the polymers used. Both the coated polymers contributed to maintaining desired lag time for colon drug release and also to releasing the medicament as per the chronobiology of the disease (Khan et al., 2018).

### Surface morphology

**Figure 6.**
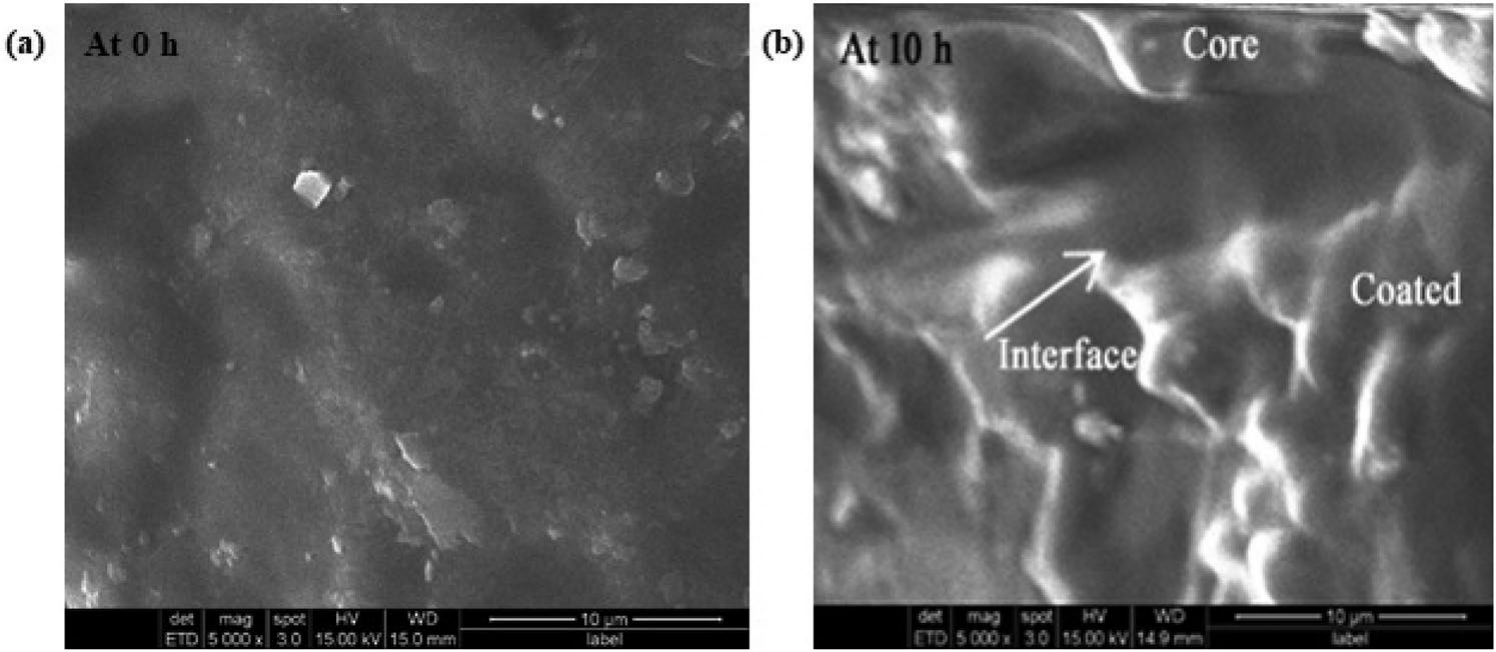
SEM images of optimized formulation at 0 h (a) and after 10 h of disintegration (b).

Optimized preparation was studied under SEM for surface topography and the results were shown in Figure 6. From this, it is noticed that the tablet facet was rough and consistent during the beginning (Figure 6a), and after 5 h the tablet showed a remarkable alteration in surface topography due to the holes and cleaves formed on the surface. It was further confirmed by a swelling test. Following the time-lapse, the inner film of the polymer coat was crumbled, utterly dissolved, and uncovered the core tablet to dissolution medium as seen in the picture taken at 10 h where the core and coat can be seen distinctly due to the interface of the two sheets in the picture (Figure 6b).

### FTIR studies

**Figure 7.**
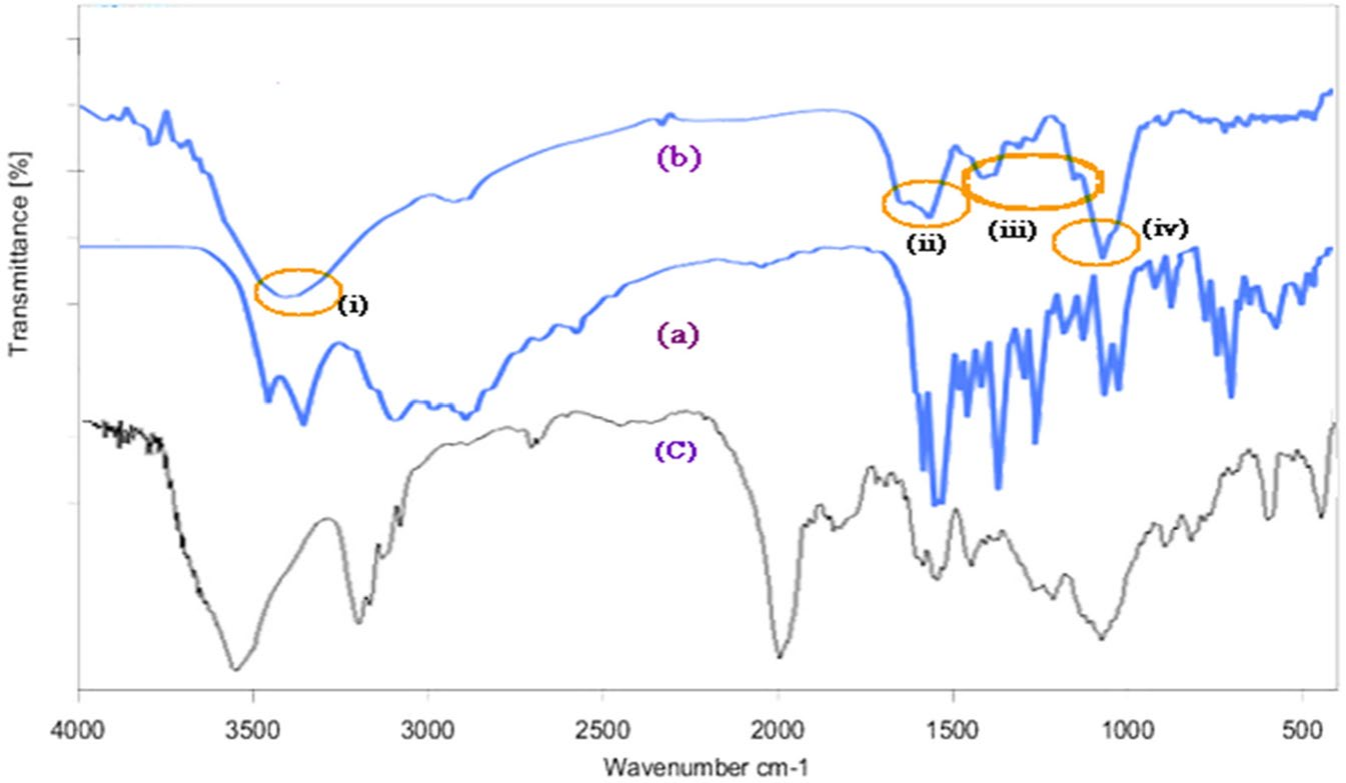
FTIR spectra of (a) Pure KT, (b) Physical mixture of optimized formulation and (c) Eudragit RLPO polymer (i) to (iv) indicates for functional groups of KT existing in both (a) and (b) confirms the compatibility of KT with selected excipients.

The FTIR spectrum of KT and optimized formulation showed similar characteristic peaks at a) 3456 cm^−1^ owing to NH2 and N-H stretching, b) 1510.68 and 1489.74 cm^−1^ corresponding aliphatic and C = C aromatic stretch, respectively, and c) 1374.5 cm^−1^ is due to C-N vibration and d) 1052.81 cm^−1^ confirms -OH bending. Other peaks at 704.54, 730.85, and 765.21 cm^−1^ confirm the C-H aromatic bending, thus confirming the compatibility of the selected excipients with KT (Figure 7).

The FTIR spectra of Eudragit RLPO (Figure 7C) show the characteristic bands of the ester groups at 1150–1190 cm^−1^ and 1240 cm^−1^ as well as the C = O ester vibration at 1730 cm^−1^.

### Stability studies

Following the six months repository, the CC-KT tablets were analyzed for lag time and percentage drug release. The average percentage difference over all time points in the amount of KT dissolved as compared to the sample at time point Zero. The fit factors for dissolution can be expressed by two approaches: f1 (the difference factor) and f2 (the similarity factor). Two dissolution profiles to be considered similar and bioequivalent, f1 should be between 0 and 15 whereas f2 should be between 50 and 10. No significant variation was found in the dissolution pattern of KT from pre and post-storage samples (Level of significance- *p* < 0.05). The *f2* similarity index of the dissolution profiles (before and after stability) was found to be 83.92, indicating no variation and a similar dissolution profile (Jacob & Nair, 2018; Zeeshan et al., 2020).

### In vivo studies

**Figure 8.**
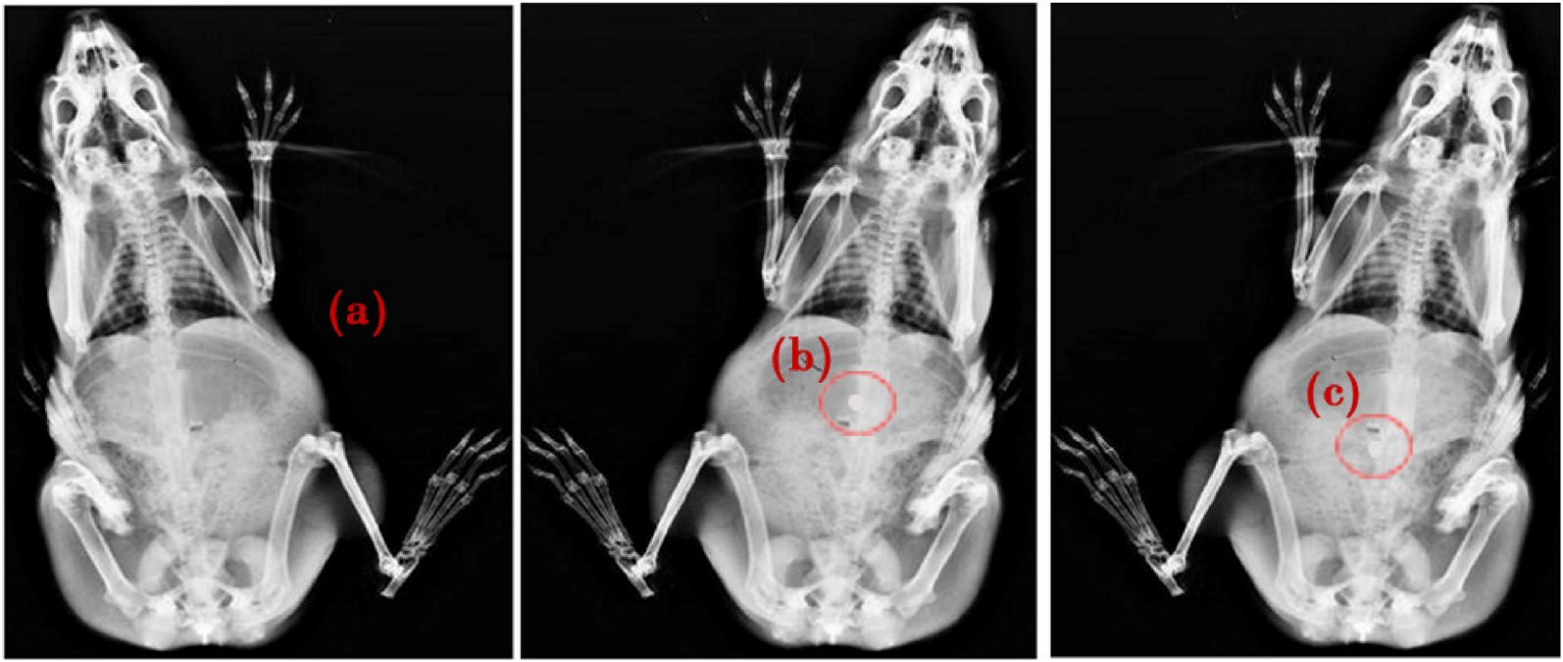
X-ray radiographs showing the location of CCT-KT tablet labeled with a radioopaque marker, Barium sulfate a) Before administration and after b) 2 h, and c) 10 h of formulation administration.

The X-ray imaging study aimed to confirm the location and behavior of the formulation in the gastrointestinal tract through the specified lag time. As shown in Figures 8b and c, the tablet remained as such through its transition from the stomach to the intestine with significant swelling. After the lag time of 10 h, the tablet got eroded slowly and contributed to the release of the loaded actives.

Twelve healthy rabbits were selected to analyze the drug concertation in plasma and other related pharmacokinetic parameters. Table 7, indicates the plasm drug concentration of Group II is relatively higher than Group I. The KT release from pure solution was quick enough, and maximum plasma concentration (Cmax-6.62 µg/mL) was reached within 1 h. Drug release from the optimized formulation was initially shown lag time of 7–8 h, where negligible drug concentration was identified. Subsequently, a rise in the drug concentration was observed and reached a maximum of 12.86 µg/mL till 12 h (Tmax). Consequently, we can confirm that plasma drug concentration was observed only after a defined lag time and followed by controlled release. This lag time will coincide with the time gap between administration of the dosage form at the night time and reaching the peak plasma concentration in the morning time when the symptoms of rheumatoid arthritis are worse. Therefore, such formulation assures availability of maximum concentration of the drug in the plasma when it is most needed, although the medication is taken at bedtime. The area under the curve (µg/mL*h) value for the developed formulation (126.24) is almost 2.5 times the drug solution (48.21). The elimination rate constant was higher for group-II animals than for group-I. Radwan et al. formulated KT nanoparticles and described the elevated plasma drug concentration up to 6 h (Radwan et al., 2010). A similar study was performed by Muhammad Suhail et al. and showed effective drug release till 24 h using hydrogel formulation of KT (Suhail et al., 2022). Conclusively, optimized compression coating enhanced the bioavailability of KT and considerably enhanced the therapeutic benefit.

**Table 7.** Pharmacokinetic profiles of pure drug solution and optimized formulation.

Parameter	Drug solution	Optimized formulation
C_max_ (µg/mL)	6.62	12.86
AUC_0-x_ (µg/mL*h)	48.21	124.26
T_max_ (h)	1	12
Ke	0.12	0.17
MRT	4	18

## Conclusions

Pulsatile KT tablets were prepared by compression coating to make the formulation with a long lag time and target-specific drug delivery. The current investigation formulated pulsatile CCT of KT with PEO WSR and Eudragit RLPO. The concentrations of coated polymers were optimized for lag time and T-95% CDR using a central composite design. The time gap of drug release from the CCT preparation can be modified by altering the PEO WSR Coagulant concentration in the outer coat. Formulation containing 174.51 mg of PEO WSR, 149.99 mg of RLPO, and a hardness of 8 kg/cm^2^ can fulfill the optimum formulation’s prerequisites. The core tablet was coated using the optimized coating formulation. *In vitro* drug release test confirms that the designed CCT-KT succeeded in maintaining desired lag time and also releasing the medicament at an appropriate rate. *In vivo* pharmacokinetic studies also assured the desired lag time and improved bioavailability. Hence the development of the CCT is a suitable method to fulfill the colon-targeted pulsatile drug release to optimize the treatment of morning stiffness in rheumatoid arthritis. The productive outcome of the current study also supports the use of PEO WSR Coagulant and RLPO polymers in designing various drug delivery techniques that help treat circadian rhythms dependent conditions.

## Author contributions

Conceptualization, N.R.N., H.M.A.; methodology, A.B.N.; software, S.M.B.; validation, H.M.A. and R.R.B.; formal analysis, H.M.A., N.R.N and R.R.B.; investigation, G.K.R.; resources, H.M.A. and N.A.A.; data curation, P.S.G.; writing—original draft preparation, G.K.R.; writing—review and editing, A.B.N. and S.M.B.; visualization, N.A.A. and S.M.B.; supervision, N.A.A. and G.K.R.; project administration, H.M.A. and S.M.B.; funding acquisition, H.M.A. All authors have read and agreed to the published version of the manuscript.

## Data Availability

Not applicable.
